# Analysis of the Curative Effect of Neoadjuvant Therapy on Pancreatic Cancer

**DOI:** 10.3389/fonc.2021.695645

**Published:** 2021-08-18

**Authors:** Liqiong Yang, Yun Bai, Qing Li, Jie Chen, Fangfang Liu, Xiechuan Weng, Fan Xu

**Affiliations:** ^1^Laboratory of Molecular Pharmacology, Department of Pharmacology, School of Pharmacy, Southwest Medical University, Luzhou, China; ^2^Department of Public Health, Chengdu Medical College, Chengdu, China; ^3^Department of Anesthesiology, Gulinxian People’s Hospital of Sichuan Province, Luzhou, China; ^4^Department of Digestive Surgery, School of Chinese Medicine, Li Ka Shing Faculty of Medicine, The University of Hong Kong, Hong Kong, Hong Kong; ^5^Department of Orthopedics, Shanghai Institute of Traumatology and Orthopaedics, Ruijin Hospital, Shanghai Jiaotong University School of Medicine, Shanghai, China; ^6^Department of Art, Art College, Southwest Minzu University, Chengdu, China; ^7^Department of Neuroscience, Beijing Institute of Basic Medical Sciences, Beijing, China

**Keywords:** pancreatic cancer, neoadjuvant chemotherapy, neoadjuvant chemo-radiotherapy, treatment, neoadjuvant therapy

## Abstract

The prevalence of pancreatic cancer is sharply increasing recently, which significantly increases the economic burden of the population. At present, the primary treatment of resectable pancreatic cancer is surgical resection, followed by chemotherapy with or without radiation. However, the recurrence rates remain high even after R0 resection. This treatment strategy does not distinguish undetected metastatic disease, and it is prone to postoperative complications. Neoadjuvant therapies, including neoadjuvant chemotherapy and radiotherapy, is being increasingly utilized in borderline resectable as well as resectable pancreatic cancer. This review summarized and discussed clinical trials of neoadjuvant therapy for pancreatic cancer, comparing resection rates, outcome measures, and adverse reactions between neoadjuvant chemotherapy and neoadjuvant chemoradiotherapy.

## Highlights

Surgery is the only potential cure for pancreatic cancer, but the survival duration of patients did not improve significantly. Pancreatic cancer has an obvious tendency to metastasize, and R0 resection is difficult to achieve. Neoadjuvant therapy is widely used, ranging from resectable pancreatic cancer, borderline resectable pancreatic cancer, and locally advanced pancreatic cancer. There are many options in neoadjuvant therapy, such as chemotherapy, radiotherapy, and chemoradiotherapy. Unfortunately, the choice of neoadjuvant treatment for pancreatic cancer remains controversial.

## Introduction

Pancreatic cancer is one of the most common malignancies of the digestive tract, and also one of the worst prognoses, with a 5-year survival rate of only 6% (1). Based on the GLOBOCAN 2020 estimates, pancreatic cancer has ranked the seventh most common cancer in the world counting 495,773 new cases and causing 466,003 deaths (4.7% of all deaths caused by cancer) in 2020 (2). In addition, the incidence and mortality of pancreatic cancer increased with age, and it is most common in men (3). The monthly medical expenses of pancreatic cancer patients are 15 times more than that of non-pancreatic cancer patients. Therefore, it is important to plan potential new therapies to manage and control patient costs (4).

## Neoadjuvant Therapy (Nat)

Frontline treatments for pancreatic cancer include surgical treatment, chemotherapy, radiation therapy, biological therapy, etc. Radical surgery is complicated and may cause more complications. Surgical treatment is local treatment, as usually the cancer tissue cannot be removed completely and it is easy to recur and metastasize. Radiation and chemotherapy use the powerful external radiation or toxic drugs to kill tumor cells in the body, unfortunately the normal cells (including immune cells) are also killed, this may induce a low immunity. Biological therapy inhibits or eliminates tumor growth by increasing the resistance of the immune system of the body to tumor cells; however, the efficiency of gene transduction is low, has poor specificity, and the efficacy of late tumors is limited (5).

Any preoperative treatment of resectable tumors, as well as treatments that may lead to surgery in the case of tumor response, are considered “neoadjuvant therapy (NAT)” (6). Unlike adjuvant therapy, NAT methods may allow the assessment of tumor response *in vivo* and improve compliance (7). The tolerance of NAT is better than that of adjuvant therapy, which can reduce the incidence of complications of pancreatic surgery. One of the most promising advantages of NAT for pancreatic cancer is that by converting the initial marginal or locally unresectable tumors into resectable tumors, it is possible to increase the number of surgical candidates. In addition, those who are converted to candidates for surgery have similar survival rates to those with initially resectable tumors (8). NAT contains neoadjuvant chemotherapy (NAC) and neoadjuvant chemo-radiotherapy (NACRT). NAC (with or without radiation therapy) is often used to reduce the staging of marginally resectable tumors and locally advanced tumors. The current evidence is mainly retrospective; however, it disclosed that NAT can increase the R0 resection rate and significantly increase the overall survival (9). Compared with NACRT, NAC appears to be equally effective in transforming the unresectable nature of resectable diseases, and it is also more effective in systemic tumor progression and overall survival (6).

Neoadjuvant radiotherapy with or without chemotherapy has a better survival than upfront surgery with or without adjuvant therapy among patients with a resectable pancreatic cancer (10). If surgery is the basis of treatment, providing pathologically negative margin (R0) resection is currently the only way to achieve the best cure rate (11). Macroscopic (R2) and microscopic (R1) marginal infiltration have similar survival trends with locally advanced or metastatic disease (12). Traditionally, R0 represents no cancer at the margins, while R1 represents microscopic disease at the margin, and R2 is representative of gross disease at the margins (seen by naked eye); see **Table 1**. For borderline resectable pancreatic cancer (BRPC), NAT could maximize the potential for an R0 resection and avoid R1/R2 resections (13). If an initially unresectable is converted to operable after NAT, microscopically complete resection has been performed (14). Resectable is the cornerstone of treatment. The ultimate goal is R0 resection. Unfortunately, even for early resectable performance, the R0 resection rate is not ideal. Therefore, it is suitable for auxiliary or neoadjuvant integrated treatment (8).

**Table 1:** Quality of surgery: RO/R1/R2.

R Designation	Gross resection	Microscopic margin
RO	Complete	negative
R1	complete	positive
R2	incomplete	positive

## Validation Method

**Figure 1:**
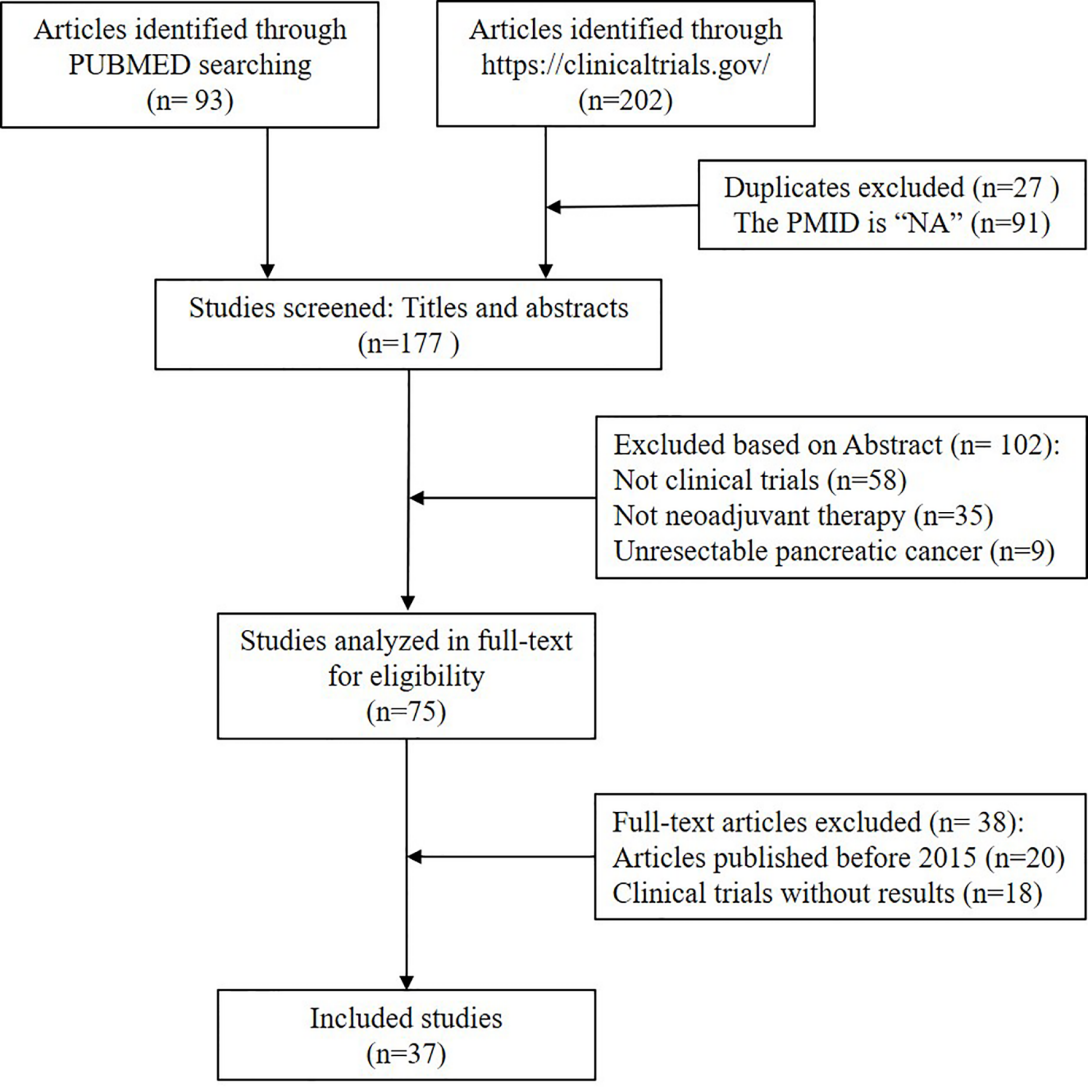
Flowchart of the included studies. Publications were retrieved by searching the following databases: PubMed (n = 93) and (https://clinicaltrials.gov/) (n = 202), with a total of 295 publications. The search strategy included keywords related to pancreatic cancer and neoadjuvant. All citations were screened to identify relevant studies, firstly, duplicate and unavailable PMID studies were excluded (27 duplicate and 91 unavailable PMID). Secondly, by the title and abstract (n = 102) and, thirdly, by full text screening (n = 38). A total of 258 publications were excluded. Finally, 37 were eligible for assessment by full paper.

We collected raw data from references, which we searched from the PUBMED with “pancreatic cancer” and “neoadjuvant” as the query terms. The article types were screened as clinical trials. A total of 93 clinical trials found since October 2020. Moreover, 202 articles were searched from (https://clinicaltrials.gov/). In total, there were 295 articles included in the study. Unresectable pancreatic cancer and irrelevant literature were further excluded. Finally, 37 clinical trials on NAT of pancreatic cancer were included in this study. The 37 records were divided into the following two tables on the basis of the type of adjuvant therapy. For a detailed reference screen plot, see **Figure 1**.

## Results

The details of the following study were extracted: first author, year of publication, interventions, study population, percentage of R0 resection after NAT, and outcome [overall survival (OS), progression-free survival (PFS), grade 3 or 4 adverse events of neutropenia, leukopenia, thrombocytopenia, and anemia].

In total, there were 18 clinical trials on patients with pancreatic cancer receiving NAC before surgery, see **Table 2**. These clinical trials recruited 9,938 patients with a resectable pancreatic cancer. The average OS was 22.87 months, and the PFS was 12.66 months. The average R0 resection rates were 73%. In detail, Mashaal et al. performed pancreaticoduodenectomy after NAT with 5-fluorouracil, leucovorin, oxaliplatin, irinotecan (FOLFIRINOX) for pancreatic ductal adenocarcinoma (PDAC) patients in 2018, the highest OS obtained was 38.7 months. The R0 resection rate was also relatively high at 84.9% (23). In 2019, Xiang et al. evaluated the effect of the modified FOLFIRINOX (mFOLFIRINOX) regimen in patients with locally advanced pancreatic cancer (LAPC) in China, they found that patients who received mFOLFIRINOX and underwent surgery had the highest PFS of 19.3 months and the higher OS of 27.7 months (19). Similarly, Marlo et al. also performed mFOLFIRINOX on patients with BRPC and LAPC. The median PFS was 18 months and a higher R0 esection rate of 86.4%, and there were no adverse reactions of neutropenia and thrombocytopenia (31). What is more, Naru et al. used gemcitabine, napaclitaxel, and S-1 NAC for patients with LAPC. It had a good R0 resection rate of 92% (25). Later, Fuyuhiko et al. assessed the feasibility and survival outcomes of NAC with gemcitabine and S1 (GS) for a PDAC planned resection. This method had a considerable R0 resection rate and OS, 91% and 30.8 months, respectively (20). Moreover, in the study of Yoshihiro et al., gemcitabine combined with Nab-paclitaxel NAC for BRPC achieved the highest R0 resection rate of 100%, with a higher OS of 27.9 months (17).

**Table 2:** Clinical trials with NAC on advanced pancreatic cancer.

Year	Author	Interventions	N	R0 resection rates	OS	PFS	Neutropenia		Leukopenia		Thrombocytopenia		Anemia	
							III	IV	III	IV	III	IV	III	IV
2020	Yoo (15)	mFOLFIRINOX followed by postoperative gemcitabine	44	–	24.7	12.2	–	–	–	–	–	–	–	–
2019	Wei (16)	gemcitabine + Erlotinib Plus Pancreaticoduodenectomy	114	59%	21.3	10.8	4	0	0	0	-	-	-	-
2019	Yoshi hiro (17)	gemcitabine plus nab-paclitaxel	31	100%	27.9	–	–	–	–	–	–	–	–	–
		upfront surgery	26	77%	12.4	–								
2019	Nagak awa (18)	NAT	297	85.7%	25.7	-	-	-	-	-	-	-	-	-
		NAC	188	84.1%	29.2	-								
		NACRT	188	87.2%	22.5	-								
		underwent upfront surgery	297	70.4%	19.0	-								
2019	Li (19)	mFOLFIRINOX	41	–	19.6	13.0	22	2	–	–	12	0	22	0
		mFOLFIRINOX and underwent surgery	14	78.6%	27.7	19.3								
		mFOLFIRINOX with nonsurgical	27	–	13.2	11.9								
		underwent upfront surgery	19	73.7%	8.9	7.6								
2019	Motoi (20)	GS	101	91%	30.8	-	35		12		3		1	
2018	Reni (21)	nab-paclitaxel combined with cisplatin, capecitabine, and gemcitabine	26	67.5%	20.7	12.5	12	8	–	–	1	0	2	0
		nab-paclitaxel followed by gemcitabine	28	44%	19.1	9.9	10	8	–	–	2	0	4	0
2018	Saito (22)	GS and LV combination	23	76.5%	21.9	11.4	8		-		0		0	
2018	Dhir (23)	FOLFIRINOX	73	84.9%	38.7	–								
		gemcitabine/nab-paclitaxel	120	80%	28.6	–								
2018	Reni (24)	adjuvant gemcitabine	26	27%	-	-	5	2	-	-	0	0	-	-
		adjuvant PEXG capecitabine	30	37%	-	-	8	4	-	-	1	0	-	-
		PEXG before and after surgery	32	63%	-	-	10	0	-	-	1	0	-	-
2017	Kondo (25)	gemcitabine/nab-paclitaxel/S-1	16	92%	–	–	1	2	2	0	0	0	0	0
2017	Okada (26)	Nab-Paclitaxel plus Gemcitabine	10	80%	-	-	2	0	5	0	2	0	1	0
2017	Mokdad (27)	NAT followed by resection	2005	–	26	–								
		upfront resection	6015	–	21	–								
2016	Okada (28)	mFOLFIRINOX with four-cycle	5	75%	-	-	2	0	0	0	0	0	0	0
		mFOLFIRINOX with eight-cycle	5	67%	-	-	1	1	1	1	1	0	0	0
2016	Katz (29)	mFOLFIRINOX	22	–	21.7	–	2	1			3	0		
2016	Masui (30)	NAC	18	-	21.7	-								
		not receive NAC	19	-	21.1	-								
2015	Blazer (31)	mFOLFIRINOX	43	86.4%	21.2	18	0	0	–	–	0	0	–	–
2015	OʼReilly (32)	gemcitabine and oxaliplatin	38	74%	27.2	-	2	0	2	0	-	-	4	0

cisplatin, epirubicin, gemcitabine, capecitabine, PEXG.

The following 20 studies clarified the results of NACRT for pancreatic cancer. From 2014 to 2020, a total of 1,030 pancreatic cancer patients were recruited, see **Table 3**. The average OS for these studies was 25.8 months, and the PFS was 18.4 months. For example, Hidetoshi et al. reported in 2019 that for resectable PDAC, NAT with gemcitabine and S-1, and 50.4 Gy of radiotherapy (GSRT) at the same time, the median survival time was as long as 55.3 months. However, there were 49 (total: 63) patients with adverse reactions of leukopenia in this regimen (38). Secondly, Janet et al. used FOLFIRINOX followed by individualized chemoradiotherapy (CRT) for borderline-resectable PDAC patients with fewer adverse events. The median OS was 37.7 months. Interestingly, among patients undergoing resection, the median PFS increased to 48.6 months with high R0 resection rates (92%) (42). Similarly, the study by Keiichi et al. also had high R0 resection rates (98%) with less adverse events. They used neoadjuvant S-1 with Concurrent hypofractionated radiotherapy in patients with resectable and borderline resectable PDAC. Even better, Jacob et al. used neoadjuvant (stereotactic body radiation therapy) SBRT Plus elective nodal irradiation (ENI) with concurrent capecitabine for resectable pancreatic cancer to obtain high R0 resection rates of 100% in 2020 (33).

**Table 3:** Clinical trials with NACRT on advanced pancreatic cancer.

Year	Author	Interventions	N	R0 resection rates	OS	PFS	Neutropenia		Leukopenia		Thrombocytopenia		Anemia	
							III	IV	III	IV	III	IV	III	IV
2020	Witt (33)	SBRT Plus ENI with Concurrent Capecitabine	13	100%	-	-	-	-	-	-	-	-	-	-
2020	Thanikac halam (34)	FOLFOX then gemcitabine and IMRT	24	-	15.1	11.9	2	1	-	-	4	0	0	0
		underwent pancreatectomy after CRT	13	84.6%	34.8	31								
2020	Tran (35)	FOLFIRINOX, radiation therapy	25	52%	24.4	13.1	10		8		-	-	2	
2019	Lin (36)	gemcitabine/leucovorin/fluorouracil/oregovomab, followed by the radiosensitizer nelfinavir	11	-	13	8.6	-	-	3	3	4	0	2	0
2019	Murphy (37)	losartan with FOLFIRINOX followed by CRT	49	61%	31.4	17.5	5	2	0	2	6	1	3	0
		underwent surgery after CRT	34	88%	33.0	31.3								
2019	Eguchi (38)	GSRT	63	85.7%	55.3	22.1	29	6	42	7	7	0	3	0
2019	Hayashi (39)	preoperative chemoradiation (50.4 Gy, S-1) followed by gemcitabine	45	95.8%	17.3	10.5	-	-	-	-	-	-	-	-
2019	Kharofa (40)	gemcitabine/nab-paclitaxel or FOLFIRINOX, then SBRT	18	92%	21	11	-	-	-	-	-	-	-	-
2018	Maurel (41)	gemcitabine and erlotinib followed by gemcitabine-erlotinib and radiotherapy	25	63.1%	23.8	12.8	-	-	-	-	-	-	-	-
2018	Murphy (42)	FOLFIRINOX, chemoradiotherapy with fluorouracil or capecitabine	48	65%	37.7	14.7	1	1	-	-	1	0	1	0
		resction operation patients	32	97%	Not reached	48.6								
2018	Jang (43)	gemcitabine-based NACRT	27	82.4%	-	-	0	0	-	-	0	0	-	-
		upfront surgery	23	33.3%	-	-	0	0	-	-	0	0	-	-
2017	Okano (44)	hypofractionated chemoradiotherapy with S-1	57	98%	16	-	2	0	2	0	0	0	0	0
2017	Mellon (45)	gemcitabine, docetaxel, and capecitabine followed by 5-fraction SBRT	81	-	37.5	17.6	-	-	-	-	-	-	-	-
2017	Nagakawa (46)	IMRT combined with gemcitabine and S-1	27	94.7%	22.4	-	-	-	-	-	-	-	-	-
2016	Roland (47)	neoadjuvant chemoradiation	222	92%	–	–								
		surgery first	85	85%	–	–								
2015	Amano (48)	GS and external beam irradiation	17	70.6%	-	-	2		-		1		1	
2015	Casadei (49)	surgery alone	20	25%	19.5	–								
		neoadjuvant chemoradiation and surgery	18	38.9%	22.4	–	–	–	4	1	1	0	0	0
2015	Golcher (50)	primary surgery	33	48%	18.9	-								
		neoadjuvant chemoradiotherapy followed by surgery	33	52%	25	-	-	-	7	2	10	1	1	1
2015	Sherman (51)	GTX, gemcitabine, and capecitabine/ radiation therapy after chemotherapy	34	58.8%	29	–	–	–	19	0	5	2	5	2
		GTX	11	72.7%	not reached									

5-fluorouracil (5-FU), leucovorin, and oxaliplatin; FOLFOX, intensity-modulated radiotherapy; IMRT, gemcitabine; docetaxel, and capecitabine, GTX.

## Advantages and Limitations Of NAT

Surgical resection first, followed by systemic chemotherapy with radiotherapy or no radiotherapy, is the current recommendation for early resectable pancreatic adenocarcinoma (27). However, these diseases may not benefit from resection because this treatment strategy fails to distinguish patients with an undetected metastatic disease and aggressive disease. Recurrence rates remain high even after R0 resections. In addition, postoperative complications associated with pancreatectomy may hinder the implementation of adjuvant therapy. Early provision of NAT is considered an alternative treatment strategy. Combining it with systemic chemotherapy and concurrent radiotherapy increased the possibility of R0 resection for patients with BRPC. NAT has many benefits, including the early treatment of micrometastatic disease and high-risk recurrence tumors, etc. (17, 52). Although NAT has many advantages, it also has some limitations. During NAT, cancer may progress locally or metastasize far away, thereby jeopardizing curative surgical treatment. NAT relies on clinical staging. Insufficient staging can lead to undertreatment, and over staging can lead to the overuse of NAT (53). In addition, there is another risk of overtreatment of cancers with a poor prognosis (54), see **Table 4**. Some scholars pointed out that patients with an early metastatic disease who are resistant to chemotherapy can be identified by preoperative systemic treatment, and the preoperative systemic treatment ensures that more patients receive multimodal treatment (55, 56). Even if NAT has a strong effect on tumors, people are worried that NAT may have an impact on the postoperative course of the disease. In fact, some studies showed that NAT of pancreatic cancer did not increase the postoperative morbidity (57). Therefore, more effective neoadjuvant programs should be applied to patients with a resectable pancreatic cancer, such as gemcitabine plus nab-paclitaxel or mFOLFIRINOX (15).

**Table 4:** The advantages and limitations of NAT.

Advantages	Limitations
-Early-treatment of micrometastasis disease, tumors with a high risk of recurrence	-Tumor progression during neoadjuvant treatment leading to missed window of opportunity for resection
-Prevent the recurrence of metastases and remove micrometastasis cells before surgery	-Relies on clinical staging and may lead to unnecessary administration of chemotherapy in over-staged patients.
-Ensures delivery of preoperative systemic therapy	-Overtreatment of tumors with a more favorable prognosis
-Improved R0 resection rate, especially in BRPC	-Delays potentially curative primary therapy
-The ability to deliver systemic therapy to all patients	-Need tissue confirmation of neoplastic process
-Less toxicity and better tolerability	
-Potential for the downstaging of borderline resectable tumors to facilitate surgical resection	
-Intact tumor vasculature not disrupted by surgery	

For NAC, the FOLFIRINOX/(m)FOLFIRINOX regimen and gemcitabine plus nab-paclitaxel is a good patient selection strategy. It is now widely recognized that NAC can achieve tumor downgrading so as to increase the surgical resection rate of pancreatic cancer, and even increase the R0 resection rate (58). It has been noted that the toxicity of the neoadjuvant FOLFIRINOX was reduced after chemoradiotherapy, with a single grade 3 toxicity of less than 10% and no toxicity-related deaths (59). In addition, the effectiveness of gemcitabine against pancreatic cancer has been widely confirmed. A large number of studies proved that the effect of single-agent chemotherapy was significantly weaker than FOLFIRINOX and multi-drug combination programs such as gemcitabine plus nab-paclitaxel, so the treatment prospects of multi-drug combination programs are good (60). The GS trial showed that GS treatment was significantly higher than gemcitabine alone (61). At the same time, judging from the incidence of adverse events and the rate of surgical resection, NAC is safe and feasible (62).

Because the surgical treatment of pancreatic cancer has a high risk of local recurrence, and radiotherapy is expected to improve the control of local diseases. Gemcitabine was chosen as the drug during concurrent radiotherapy due to its well-known radio sensitizing properties (63). A clinical trial evaluated the effects of gemcitabine-based NACRT. Compared with upfront surgery, patients who received gemcitabine-based NACRT showed a benefit from OS (17.1 months *vs*. 13.5 months) and an increased R0 resection rate (65% *vs*. 31%) (64). The use of full-dose GSRT for NAT of resectable PDAC uncovered the outstanding clinical efficacy and acceptable tolerability, and achieved a low local recurrence rate (38). Yuichi et al. demonstrated that IMRT combined with gemcitabine and S-1 can be used as NACRT for patients with a resectable pancreatic cancer with low gastrointestinal toxicity. IMRT can provide a more effective NACRT through powerful chemotherapy drugs (46). As a component of NAT, SBRT has a good safety and tolerability (65). The advantages of SBRT are that it reduces the treatment time and can accurately locate the target area, but the disadvantage is that it does not provide the opportunity to selectively kill tumor cells using radio sensitizing chemotherapy (35).

What is more, clarifying the tumor characterization before the surgery or chemotherapy is of great importance. For example, several scientific society including Okusaka et al. (66), Dumonceau et al. (67), Jenssen et al. (68), and Eloubeidi et al. (69) recommended to use the EUS guided tissue acquisition before surgery and neoadjuvant chemotherapy, therefore the most appropriate treatment therapy may be approached soon.

## Conclusion and Prospection

NAT improved the OS and PFS time of patients with a resectable pancreatic cancer compared with upfront surgery. The combination of multidisciplinary NAT with systemic chemotherapy and concurrent radiotherapy increases the possibility of R0 resection for patients with a resectable marginal pancreatic cancer. Judging from the incidence of adverse events and the rate of surgical resection, NAC is safe and feasible. In short, NAT significantly improved the R0 resection rate and sufficient survival duration. NAC and NACRT provide oncological benefits for patients with BRPC. However, the choice of pancreatic cancer NAT regimen, drug dosage, timing of administration, and drug cycle also need further research. How to select patients who are suitable for NAT and formulate the most optimized NAT solution will be a problem that we urgently need to solve. The ultimate goal of scientists is to allow more patients with a resectable pancreatic cancer to benefit from NAT in order to improve their prognosis. NAT is one of the major advances in multidisciplinary oncology in the past few decades, which requires a multidisciplinary treatment team and the best infrastructure for complex oncology care.

## Author Contributions

LY and FX wrote the paper. XW and JC collected data from the reference. YB and QL collected the data. FL drew the figure. All authors contributed to the article and approved the submitted version.

## Funding

This work was supported by the Chengdu Medical College Foundation (CYZ19-33), Chengdu Science and Technology Bureau focuses on research and development support plan (2019-YF09-00097-SN), the popular scientific research project of Sichuan Health Commission (20PJ171), and Sichuan undergraduate innovation and startup program funding support (S201913705080, S201913705130, S201913705059, S202013705070, S202013705075, S202013705108), and Yunnan education program (SYSX202036). National Natural Science Foundation of China (82073833) and Southwest University for Nationalities special fund for basic scientific research operations of central universities (2021NYB08).

## Conflict of Interest

The authors declare that the research was conducted in the absence of any commercial or financial relationships that could be construed as a potential conflict of interest.

## Publisher’s Note

All claims expressed in this article are solely those of the authors and do not necessarily represent those of their affiliated organizations, or those of the publisher, the editors and the reviewers. Any product that may be evaluated in this article, or claim that may be made by its manufacturer, is not guaranteed or endorsed by the publisher.
